# Immune tolerance induction using the thyrotropin receptor epitope 78–94 (p37) prevents Graves’ disease in HLA-DR3 transgenic mice

**DOI:** 10.3389/fimmu.2025.1633350

**Published:** 2025-11-03

**Authors:** Hidefumi Inaba, Itsuki Nonaka, Daiki Hashimoto, Moritoshi Hirono, Shuhei Morita, Hiroaki Kimura, Hiroshi Iwakura, Takashi Akamizu, Masanori Nakata

**Affiliations:** ^1^ Department of Physiology, Wakayama Medical University, Wakayama, Japan; ^2^ The First Department of Medicine, Wakayama Medical University, Wakayama, Japan; ^3^ Department of Pharmaceutical Health Sciences, Kyushu University of Medical Sciences, Nobeoka, Japan; ^4^ Department of Pharmacotherapeutics, School of Pharmaceutical Science, Wakayama Medical University, Wakayama, Japan; ^5^ Department of Internal Medicine, Kuma Hospital, Kobe, Japan

**Keywords:** Graves’ disease, thyrotropin receptor, HLA-DR, regulatory T cells, tolerance

## Abstract

Graves’ disease (GD) is an organ-specific autoimmune thyroid disorder characterized by anti–thyrotropin receptor (TSH-R) antibodies (TRAb), with strong genetic susceptibility conferred by the HLA-DRB1*03:01 (DR3) allele. We investigated whether pre-immunization with the immunodominant TSH-R–derived peptide spanning residues 78–94 (ISRIYVSIDVTLQQLES; p37) could induce immune tolerance and prevent GD in DR3 transgenic mice. GD was induced by intramuscular injection of adenovirus encoding human TSH-R (Ad-TSH-R289). Mice were pretreated with p37 either as a single 50 μg dose or by step-up escalation protocol (0.05 μg, 0.5 μg, and 5 μg), with or without a final 50 μg dose. Ad-TSH-R289 immunization was performed in all groups three weeks after the final peptide administration. While the single-dose protocol failed to prevent disease, the step-up protocol, particularly when including the final 50 μg dose, significantly suppressed serum free thyroxine (FT4) and TRAb levels and prevented histopathological changes in the thyroid gland. These effects were accompanied by an increase in splenic regulatory T cells (CD4^+^CD25^+^FoxP3^+^), a reduction in CD4^+^PD-1^+^ T cells, and an increase in CD8^+^PD-1^+^ T cells. Depletion of Tregs using an anti-CD25 antibody abrogated the protective effect and elevated serum IFN-γ levels, underscoring the essential role of Tregs in mediating tolerance. In contrast, the weakly immunogenic variant of p37 (37m) provided limited protection, underscoring the necessity of the native peptide sequence. In conclusion, these findings demonstrate that step-up immunization with p37 induces antigen-specific immune tolerance and effectively prevents the development of GD in HLA-DR3 transgenic mice. This strategy represents a promising approach for antigen-specific immunotherapy in autoimmune thyroid disease.

## Introduction

Graves’ disease (GD) is an organ-specific autoimmune disorder characterized by anti-thyrotropin receptor (TSH-R) autoantibodies (TRAb) (1–3). The prevalence of GD is approximately 1.2%, with a higher incidence in females than in males, with peak onset between the ages of 30 and 50 (1). Both genetic and environmental factors contribute to the breakdown of immune tolerance to self TSH-R, leading to the onset of GD (1–3). Genetic factors account for approximately 70–80% of disease susceptibility (4). Predisposing genes include *HLA*, *CTLA-4*, *TSHR*, *PTPN22*, *CD40*, and *FOXP3* (5). Among these, inheritance of the HLA-DRB1*03:01 (DR3) allele shows strong association with GD (1–3).

The A subunit of TSH-R exists partially in a shed, soluble form (3). Rapoport et al. demonstrated that immunization of mice with the human TSH-R A subunit induces GD in mice (6). The TSH-R A subunit, once shed into the circulation, is taken up by antigen-presenting cells via endocytosis, processed into peptides within endosomes, and subsequently presented on major histocompatibility complex (MHC) class II molecules such as HLA-DR to CD4^+^ T cells, thereby initiating an autoimmune response (3).

Based on data obtained from HLA-DR3 transgenic mice and human clinical studies, we identified several immunogenic TSH-R peptides (7–9). Among them, the peptide spanning residues 78–94 (ISRIYVSIDVTLQQLES), referred to as p37, exhibited the highest immunogenicity. A mutated variant, 37m (ISRIYVSIDATLSQLES), containing amino acid substitutions at positions 5 and 8, was engineered to reduce immunogenicity and indeed demonstrated significantly diminished immune reactivity (10).

Although approximately 50% of patients with GD achieve remission after 12–18 months of antithyroid drug therapy, relapse is common, and adverse effects—such as leukopenia—remain a significant concern (11). Radioiodine (I-131) therapy is often suboptimal and may lead to recurrent GD or hypothyroidism, whereas total thyroidectomy necessitates general anesthesia and lifelong thyroid hormone replacement therapy (1, 12). Consequently, novel therapeutic strategies are warranted, including interventions targeting TRAb production or activity, the development of small-molecule TSH-R antagonists, and antibody-based therapies (12).

Impairments in both central (thymic) and peripheral immune tolerance mechanisms are implicated in the pathogenesis of GD (13). In particular, a reduction in the number and functional capacity of regulatory T cells (Tregs) has been associated with the breakdown of peripheral tolerance (14–16). The therapeutic induction of immune tolerance has been demonstrated in several autoimmune disease models—for example, administration of myelin basic protein (MBP) has been shown to suppress disease onset in experimental autoimmune encephalomyelitis (17), while insulin peptide administration prevents the development of type 1 diabetes (18). In earlier studies, oral administration of thyroglobulin (Tg) was reported to suppress experimental autoimmune thyroiditis (19). The aim of the present study was to investigate whether pre-immunization with TSH-R peptides prior to disease induction could establish immune tolerance in a murine model of GD and to elucidate the underlying immunological mechanisms.

## Materials and methods

### Mice

Transgenic mice expressing the human HLA-DRB1*03:01 (DR3) allele, kindly provided by Dr. Chella David (Mayo Clinic), were generated by co-injection of HLA-DRA and HLA-DRB1*03:01 genes into (C57BL/6 × DBA/2) F1 × C57BL/6 embryos backcrossed onto a B10 genetic background (20). These mice express the human HLA-DR3 transgene and lack endogenous mouse MHC class II molecules. Age-matched female mice (5–8 weeks old) were used for all experiments. Animals were housed under conventional conditions. All animal care and experimental procedures were conducted in accordance with the Guidelines for Animal Experimentation of Wakayama Medical University and were approved by the Institutional Animal Care and Use Committee.

### Peptide synthesis

A known GD-associated epitope peptide corresponding to amino acid residues 78–94 of the human TSH-R (ISRIYVSIDVTLQQLES), referred to as p37, was synthesized (8, 9). A mutated version of this peptide, termed 37m (ISRIYVSIDATLSQLES), containing two substitutions at positions critical for DR3 binding (position 5: Valine to Alanine; position 8: Glutamine to Serine), was also generated as previously described, to reduce binding affinity to the T cell receptor (10). Additionally, an HLA-DR3 high-affinity binder peptide (TSH-R 132–150, termed p10: GIFNTGLKMFPDLTKVYST) and a control peptide (TSH-R 109–124, termed p8: RNTRNLTYIDPDALKE) were included in the study (**Table 1**). Peptide sequences were confirmed and purity (>90%) was verified by reverse-phase HPLC (Peptide Institute, Inc., Osaka, Japan and CHI scientific, Inc., Maynard, MA, USA).

**Table 1:** TSH-R derived peptides used in the current study.

Name	Position	AA	IC_50_ for HLA-DR3^*1^	Clinical T-cells stimulation^*2^	Notes
p37	78–94	ISRIYVSIDVTLQQLES	0.3	N/A	Strong epitope for HLA-DR3 refs (7–9)
37m	78–94, but position 88 and 91 were altered	ISRIYVSIDATLSQLES	0.3	N/A	A mutated peptide of p37 ref (10)
p10	132-150	GIFNTGLKMFPDLTKVYST	0.67	2	A strong binder to HLA-DR3 refs (7, 9)
p8	109-124	RNTRNLTYIDPDALKE	5.5	0	Control peptide refs (7, 9)

AA, amino acid sequence. ^*1^In vitro binding affinity to HLA-DR3 (IC50 in micromoles) (refs 7-9). ^*2^Clinical T-cells stimulation scores ref (7).

N/A, not applicable.

### Propagation of adenovirus vector encoding human TSH-R 1-289

A recombinant adenovirus expressing the extracellular domain of human TSH receptor (amino acids 1–289; Ad-TSH-R289) was generated for GD induction. PCR was performed using the pSVL plasmid encoding full-length human TSH-R (1–764), kindly provided by Dr. Refetoff (University of Chicago). The primers used were: Forward: 5′-CACCATGAGGCCGGCGGACTTG-3′ and Reverse: 5′-TTACTGATTCTTAAAAGCACAGCA-3′. PCR amplification was followed by cloning into the pENTR/D-TOPO vector, and subsequent recombination into the pAd/CMV/V5-DEST vector using the Gateway system (Thermo Fisher Scientific, Waltham, MA, USA). The constructed plasmid was transfected into 293A cells, and a single adenoviral plaque was isolated and expanded. A control adenovirus lacking the TSH-R insert: pAd/CMV/V5-DEST was similarly prepared and used as a control. Viral titers: plaque forming unit (PFU) were determined by OD260 or TCID_50_ assays. Adenoviruses were purified by CsCl gradient ultracentrifugation and dialyzed in PBS (8). Expression of TSH-R (1–289) was confirmed by Western blot using a monoclonal anti–TSH-R antibody (clone A10, Advanced Targeting Systems, San Diego, CA).

### Induction of GD in HLA-DR3 transgenic mice and tolerance induction

**Figure 1:**
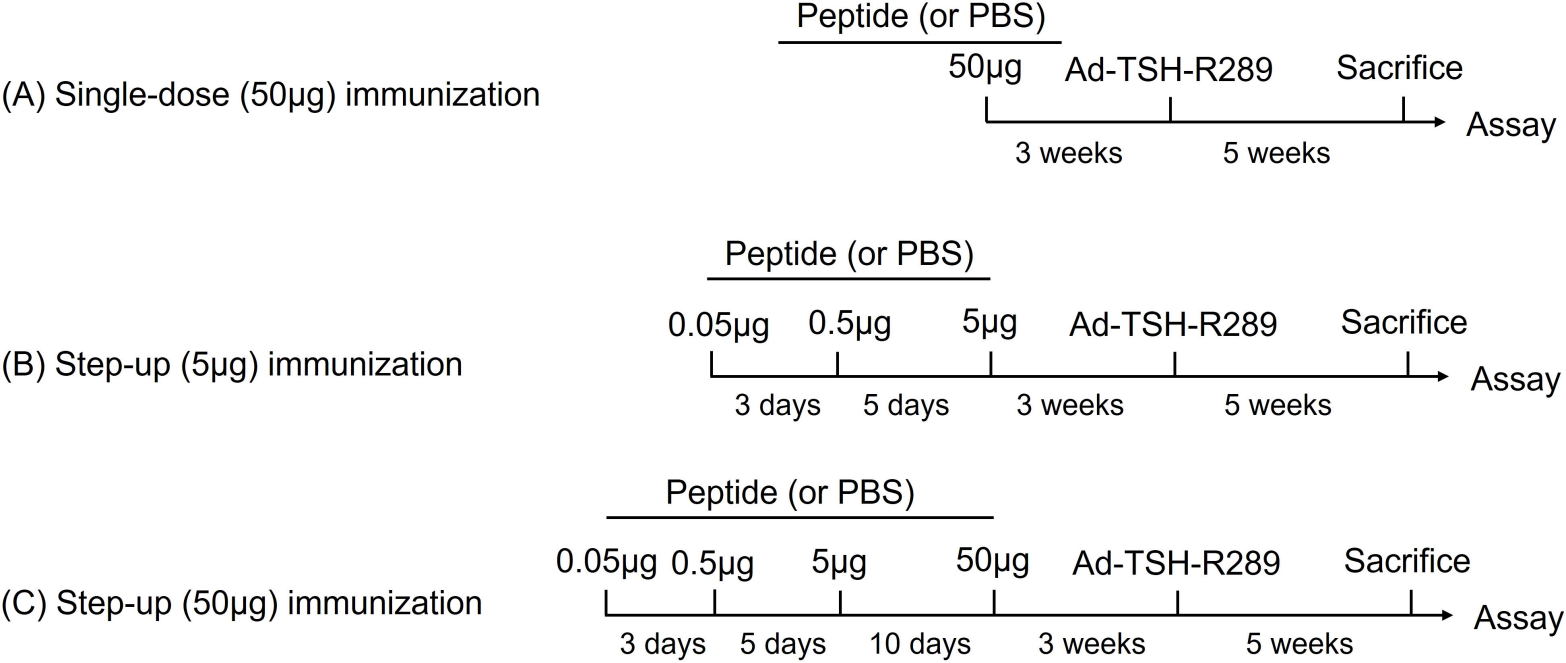
Tolerance induction protocols. **(A)** Single-dose immunization with 50 μg of peptide. **(B)** Step-up immunization protocol escalating to 5 μg of peptide. **(C)** Step-up immunization protocol escalating to 50 μg of peptide. Mice were immunized subcutaneously with the indicated peptides three weeks prior to adenoviral administration of TSH-R289.

GD was induced by intramuscular injection of 100 μL phosphate-buffered saline (PBS) containing 1 × 10^9^ particles of Ad-TSH-R289 into the quadriceps muscle. As a control, the same amount of Ad-CMV-DEST was administered. Three weeks prior to Ad-TSH-R289 immunization, mice received subcutaneous pretreatment with TSH-R peptide p37, 37m, p10, p8, or PBS. Two immunization protocols were employed: a single-dose protocol and a step-up protocol (**Figure 1**). In the single-dose protocol, mice were subcutaneously injected with 50 μg of each peptide dissolved in 50 μL PBS (**Figure 1A**). In the step-up protocol, mice received subcutaneous injections of each peptide in an escalating dose regimen: 0.05 μg on day 0, 0.5 μg on day 3, 5 μg on day 8, and 50 μg on day 18, for a total of four injections (**Figures 1B, C**). All peptides were dissolved in 50 μL PBS. All mice were sacrificed five weeks after Ad-TSH-R289 immunization, and serum, thyroid glands, and spleens were collected for analysis.

### Treg depletion treatment

To deplete Tregs, a subgroup of mice received 500 µg of anti-CD25 antibody (clone PC61.5, Rat IgG1λ; eBioscience, San Diego, CA, USA) intraperitoneally 4 days prior to Ad-TSH-R289 immunization.

### Free thyroxine (FT4) and anti-TSH-R antibody measurements

Serum FT4 levels were measured using an ELISA kit (ENZAPLATE-FT4, Siemens Healthcare Diagnostics, Tokyo, Japan; normal range: 0.79–2.00 ng/dL). Anti–TSH-R antibodies were measured using a third-generation TRAb assay (Tosoh Corporation, Tokyo, Japan; normal range: <2.0 IU/L).

### Enzyme-linked immunosorbent assay of mouse serum cytokines

Mouse serum IL-2, IL-4, IL-10, and IFN-γ levels were measured using ELISA kits (Thermo Fisher Scientific, Waltham, MA, USA). Serum (12.5 μL) was diluted to 100 μL with assay diluent and processed according to the manufacturer’s instructions.

### Flow cytometry

Splenocytes were stained with the following antibodies: CD4-FITC (RM4-5), CD8-PE (53-6.7), IFN-γ–PerCP-Cy5.5 (XMG1.2), IL-4–PE (11B11), FoxP3–PE-Cy5 (FJK-16s), CD44-PE (IM7), CD62L–PerCP-Cy5.5 (Mel-14), CD25-PE (PC61.5), and PD-1–PE-Cy7 (J43). Intracellular staining for FoxP3 was performed using the FoxP3/Transcription Factor Staining Buffer Set according to the manufacturer’s protocol (eBioscience, San Diego, CA, USA). Samples were analyzed using a FACSCalibur™ (Becton Dickinson, New Jersey, USA).

### Cell proliferation assay

Splenocytes were isolated on sacrifice as described previously (7–9). Splenocytes (5 × 10^5^ cells/well) were cultured in 96-well round-bottom plates in RPMI 1640 medium supplemented with 25 mM HEPES, 2 mM L-glutamine, 100 U/mL penicillin, 100 µg/mL streptomycin, and 10% FCS (Gibco BRL). Cells were incubated for 3 days with or without TSH-R peptides (5 µg/mL). BrdU was added during the final 8 hours, and incorporation was measured using a chemiluminescent assay (Roche Diagnostics, Mannheim, Germany). Results were expressed as a stimulation index (SI): BrdU uptake in peptide-stimulated wells divided by uptake in unstimulated wells.

### Histological examinations

Thyroid glands and orbital tissues were fixed in 10% formalin and embedded in paraffin at Applied Medical Research (Osaka, Japan). Sections (5 μm) were stained with hematoxylin and eosin.

### Statistical analysis

Data were analyzed using Mann–Whitney U tests for comparisons between two groups. For comparisons among multiple groups, the Kruskal–Wallis test was used. Spearman’s rank correlation coefficient (Rs) was used to assess correlations. All analyses were performed using JMP software version 17 (SAS Institute Inc.). A P-value <0.05 was considered statistically significant.

## Results

### Induction of mice GD in HLA-DR3 transgenic mice

Following immunization with Ad-TSH-R289, a subset of mice with elevated serum FT4 levels (>2.0 ng/dL) and positive for TRAb (>2.0 IU/L) showed histopathological features consistent with Graves’ disease (GD), including thyroid enlargement, colloid ballooning, colloid vacuolization, and thyrocyte hyperplasia (**Supplementary Figures 1A, B**). Among a total of 54 mice, 11 (20%) had elevated FT4 levels and 19 (35%) tested positive for TRAb (**Supplementary Table 1**). In contrast, mice immunized with the control adenovirus vector, Ad-CMV-DEST, did not show these changes (**Supplementary Figures 2A, B**).

### Tolerance induction with TSH-R peptides

**Figure 2:**
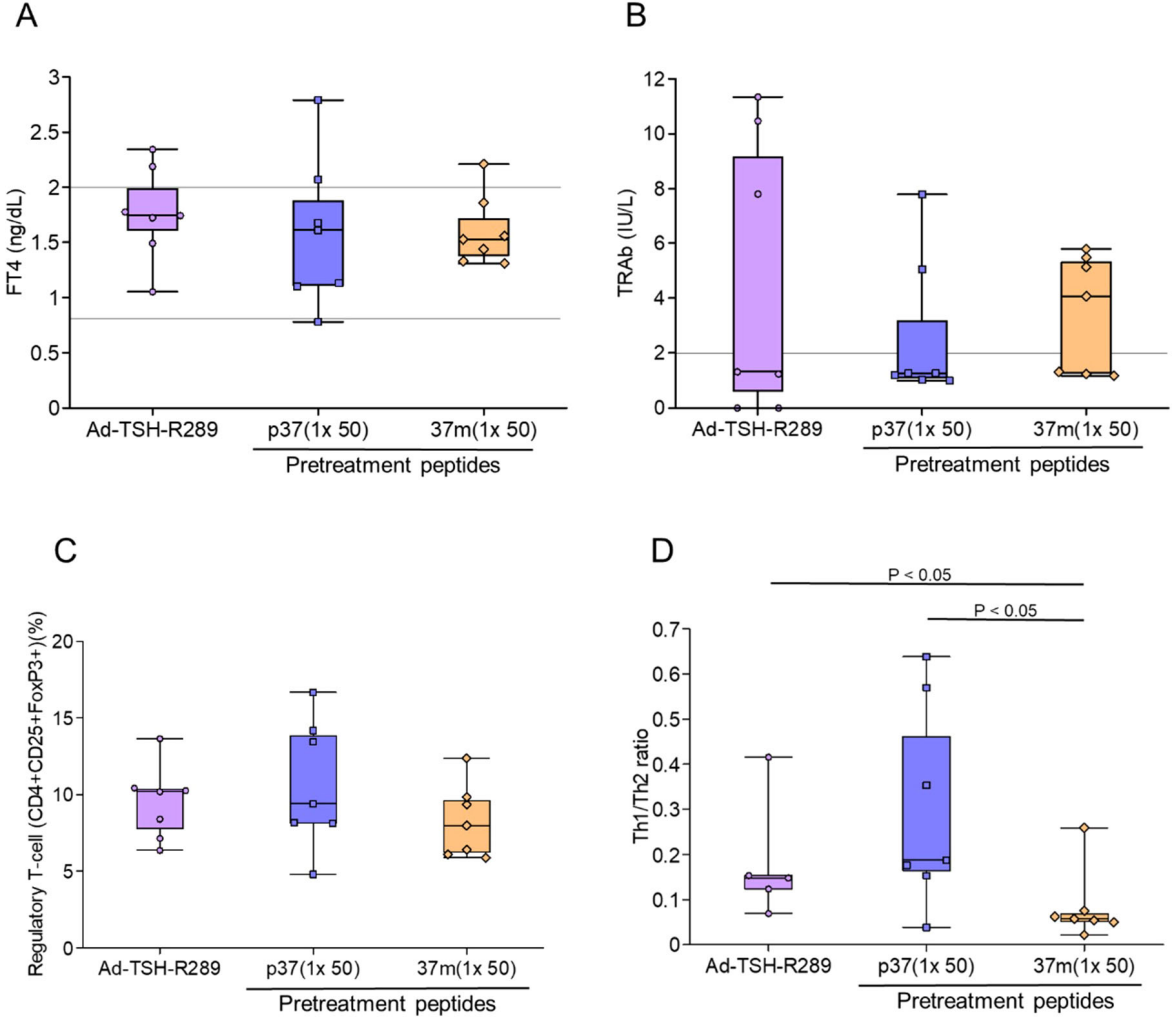
Effects of single-dose peptide immunization. **(A)** Serum FT4 levels, **(B)** TRAb levels, **(C)** frequency of splenic Tregs (CD4^+^CD25^+^FoxP3^+^), and **(D)** Th1/Th2 cytokine ratio were assessed at the time of sacrifice, five weeks after Ad-TSH-R289 immunization. Mice were pretreated with a single subcutaneous dose (50 μg) of peptide p37, 37m, or PBS. A significantly higher Th1/Th2 ratio was observed in the p37 group compared to the other groups (*p* < 0.05). “p37 (1×50)” and “37m (1×50)” represent single-dose immunizations with 50 μg of each peptide. Reference ranges: FT4, 0.79–2.00 ng/dL; TRAb, <2.0 IU/L (indicated by horizontal lines).

Pretreatment with a single dose of TSH-R peptides did not result in significant changes in serum FT4 levels, TRAb titers, or the proportion of splenic Tregs (CD4^+^CD25^+^FoxP3^+^) compared to the Ad-TSH-R289 group (**Figures 2A–C**) (**Supplementary Figures 3A, B**). Interestingly, the Th1/Th2 cytokine ratio was higher in the p37 group than in both the Ad-TSH-R289 and 37m groups (**Figure 2D**). Correlation analysis revealed that TRAb levels positively correlated with the proportions of naïve (CD4^+^CD44^low^CD62L^high^) and memory (CD4^+^CD44^high^CD62L^high^) T cells, and inversely correlated with the frequencies of Tregs and effector T cells (CD4^+^CD44^high^CD62L^low^) (**Table 2A**). Conversely, Treg frequency was positively associated with the Th1/Th2 ratio, effector T cells, and serum IFN-γ levels, and negatively associated with naïve and memory T cells (**Table 2A**).

**Table 2A:** Correlations among FT4 or TRAb levels, splenocyte subsets, and serum cytokine levels in a one-dose immunization.

	Th1/Th2 ratio		Regulatory T-cells (CD4^+^CD25^+^ FoxP3^+^)(%)		Naïve T-cells (CD4^+^CD44^low^ CD62L^high^)(%)		Memory T-cells (CD4^+^CD44^high^ CD62L^high^)(%)		Effector T-cells (CD4^+^CD44^high^ CD62L^low^)(%)		IL-2 (pg/mL)		IL-4 (pg/mL)		IL-10 (pg/mL)		IFN-γ (pg/mL)	
	^*1^Rs	^*2^P	^*1^Rs	^*2^P	^*1^Rs	^*2^P	^*1^Rs	^*2^P	^*1^Rs	^*2^P	^*1^Rs	^*2^P	^*1^Rs	^*2^P	^*1^Rs	^*2^P	^*1^Rs	^*2^P
FT4 (ng/dL)	-0.345	0.148	-0.075	0.759	0.2678	0.268	0.1404	0.567	-0.235	0.333	-0.203	0.4043	-0.043	0.861	0.089	0.716	-0.047	0.849
TRAb (IU/L)	-0.254	0.295	**-0.534**	**0.019**	**0.6454**	**0.003**	**0.5795**	**0.009**	**-0.608**	**0.006**	0.0665	0.7867	0	1	0.082	0.739	-0.405	0.085
Regulatory T-cells (%)	**0.546**	**0.016**	N/A	N/A	**-0.819**	**<.0001**	**-0.846**	**<.0001**	**0.8474**	**<.0001**	0.0714	0.7715	0.215	0.376	-0.149	0.543	**0.5791**	**0.009**

^*1^Spearman’s rank correlation coefficient ^*2^P-value.

N/A, not applicable. P-values < 0.05 are shown in bold.

**Figure 3:**
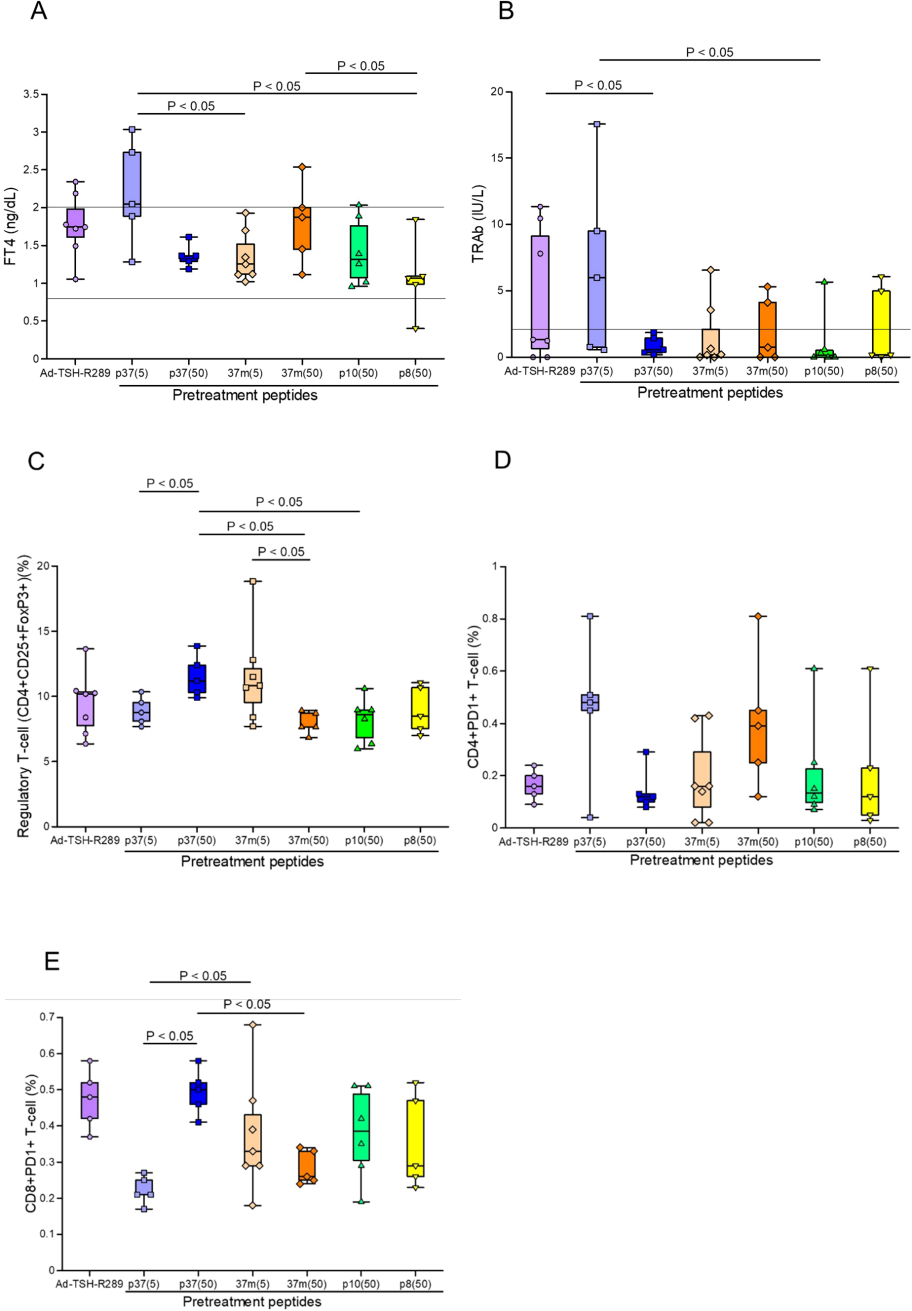
Effects of step-up peptide immunization on splenic immune cell populations. **(A)** Serum FT4 levels, **(B)** TRAb levels, **(C)** frequency of splenic Tregs (CD4^+^CD25^+^FoxP3^+^), **(D)** proportion of CD4^+^PD-1^+^ T cells, and **(E)** proportion of CD8^+^PD-1^+^ T cells were assessed five weeks after Ad-TSH-R289 immunization. Mice were pretreated using a step-up protocol with peptide p37 (5 or 50 μg), 37m (5 or 50 μg), p10 (50 μg), or p8 (50 μg). The p37 (50 μg) group exhibited significant suppression of FT4 and TRAb levels, a higher frequency of Tregs, a lower proportion of CD4^+^PD-1^+^ T cells, and an increased proportion of CD8^+^PD-1^+^ T cells compared to the other groups (*p* < 0.05). “p37 (5)” and “37m (5)” indicate step-up immunizations escalating to 5 μg; “p37 (50)”, “37m (50)”, “p10 (50)”, and “p8 (50)” indicate step-up immunizations escalating to 50 μg. Reference ranges: FT4, 0.79–2.00 ng/dL; TRAb, <2.0 IU/L (indicated by horizontal lines).

To enhance the efficacy of peptide-based tolerance induction, a step-up dosing protocol was evaluated. Among the experimental groups, mice receiving step-up administration of p37 up to 50 μg exhibited the most effective suppression of FT4 elevation, with levels maintained within the normal range (**Figure 3A**). This group also showed greater suppression of TRAb titers compared to the group receiving p37 up to 5 μg (**Figure 3B**). Additionally, the p37 (up to 50 μg) group demonstrated a more pronounced expansion of splenic Tregs (**Figure 3C**), a reduced frequency of CD4^+^PD-1^+^ T cells (**Figure 3D**), and an increased frequency of CD8^+^PD-1^+^ T cells (**Figure 3E**). In the step-up protocol, FT4 levels positively correlated with the frequencies of CD4^+^PD-1^+^ and naïve T cells, and negatively with CD8^+^PD-1^+^ T cells (**Table 2B**). Similarly, TRAb titers showed positive correlations with CD4^+^PD-1^+^ and naïve T cells, and negative correlations with CD8^+^PD-1^+^ T cells and Tregs. In contrast, Treg frequency was positively associated with CD8^+^PD-1^+^ T cells, and inversely correlated with CD4^+^PD-1^+^ and naïve T cells (**Table 2B**).

**Table 2B:** Correlations among FT4 or TRAb levels, splenocyte subsets, and serum cytokine levels in a step-up immunizations.

	CD4^+^PD1^+^ T-cells (%)		CD8^+^PD1^+^ T-cells (%)		Regulatory T-cells (CD4^+^CD25^+^ FoxP3^+^)(%)		Naïve T-cells (CD4^+^CD44^low^CD62L^high^)(%)		Memory T-cells (CD4^+^CD44^high^ CD62L^high^)(%)		Effector T-cells (CD4^+^CD44^high^ CD62L^low^)(%)		IL-2 (pg/mL)		IL-4 (pg/mL)		IL-10 (pg/mL)		IFN-γ (pg/mL)	
	^*1^Rs	^*2^P	^*1^Rs	^*2^P	^*1^Rs	^*2^P	^*1^Rs	^*2^P	^*1^Rs	^*2^P	^*1^Rs	^*2^P	^*1^Rs	^*2^P	^*1^Rs	^*2^P	^*1^Rs	^*2^P	^*1^Rs	^*2^P
FT4 (ng/dL)	**0.417**	**0.004**	**-0.294**	**0.048**	-0.289	0.051	**0.3422**	**0.02**	-0.148	0.326	0.0904	0.5503	-0.067	0.662	-0.133	0.384	0.0221	0.885	-0.082	0.592
TRAb (IU/L)	**0.489**	**6E-04**	**-0.469**	**0.001**	**-0.35**	**0.017**	**0.523**	**2E-04**	0.1548	0.304	-0.228	0.127	-0.057	0.712	-0.2	0.187	-0.004	0.979	-0.244	0.107
Regulatory T-cells (%)	**-0.474**	**9E-04**	**0.5411**	**1E-04**	N/A	N/A	**-0.604**	**<.0001**	0.049	0.746	0.1007	0.5057	0.164	0.281	-0.054	0.724	-0.038	0.803	0.1048	0.493

^*1^Spearman’s rank correlation coefficient ^*2^P-value

N/A, not applicable. P-values < 0.05 are shown in bold.

**Figure 4:**
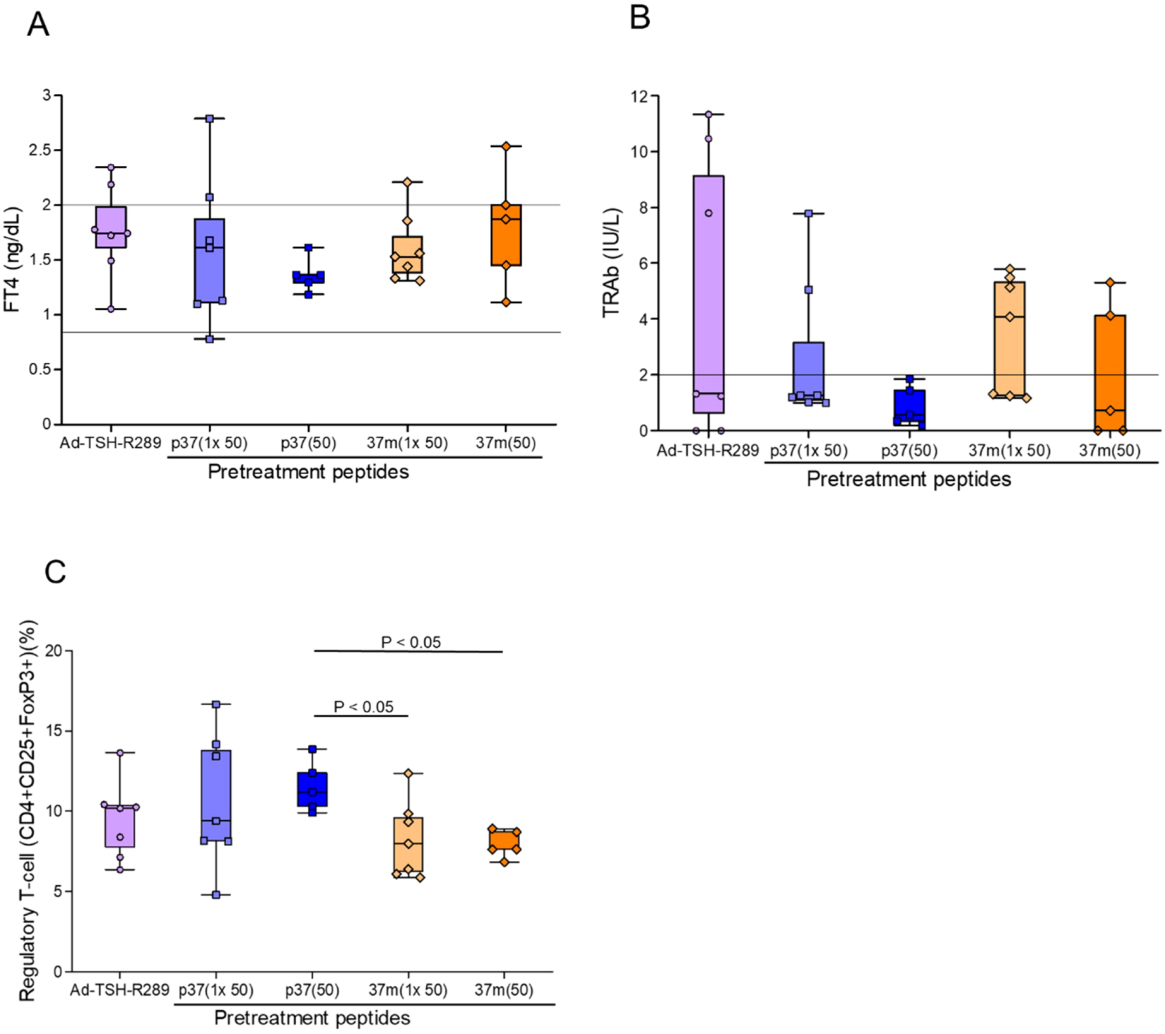
Comparison of peptide immunization protocols on thyroid function and Treg induction. **(A)** Serum FT4 levels, **(B)** TRAb levels, and **(C)** frequency of splenic Tregs (CD4^+^CD25^+^FoxP3^+^) were assessed at the time of sacrifice, five weeks after Ad-TSH-R289 immunization. Mice were pretreated with either a single-dose (50 μg) or a step-up (up to 50 μg) immunization protocol using peptide p37 or 37m. Although the differences did not reach statistical significance, the step-up p37 group tended to show greater suppression of FT4 and TRAb levels. Moreover, step-up p37 immunization more effectively induced Treg expansion compared to the single-dose groups. “p37 (1×50)” and “37m (1×50)” represent single-dose immunizations with 50 μg of each peptide, while “p37 (50)” and “37m (50)” represent step-up immunizations escalating to 50 μg. Reference ranges: FT4, 0.79–2.00 ng/dL; TRAb, <2.0 IU/L (indicated by horizontal lines).

Finally, the efficacy of step-up versus single-dose peptide immunization was compared. Although the differences did not reach statistical significance, the step-up protocol with p37 up to 50 μg consistently demonstrated superior control of serum FT4 levels and TRAb suppression (**Figures 4A, B**). This group also showed a more robust increase in the frequency of splenic Tregs (**Figure 4C**).

### Treg depletion treatment

**Figure 5:**
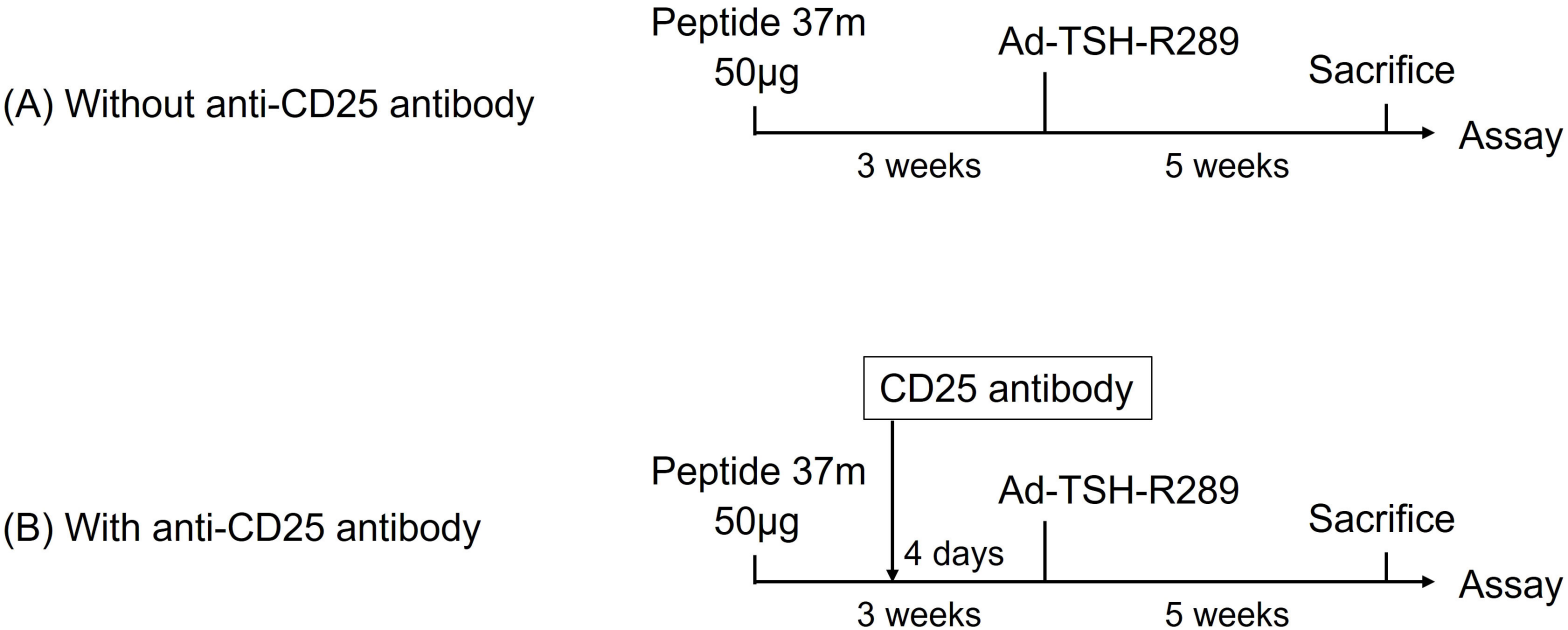
Treg depletion using anti-CD25 antibody **(A)** Control group pretreated with peptide 37m followed by Ad-TSH-R289 immunization, without anti-CD25 antibody administration. **(B)** Experimental group pretreated with peptide 37m and administered anti-CD25 antibody four days prior to Ad-TSH-R289 immunization. Splenic Treg depletion was confirmed by flow cytometric analysis of splenocytes at the time of sacrifice.

**Figure 6:**
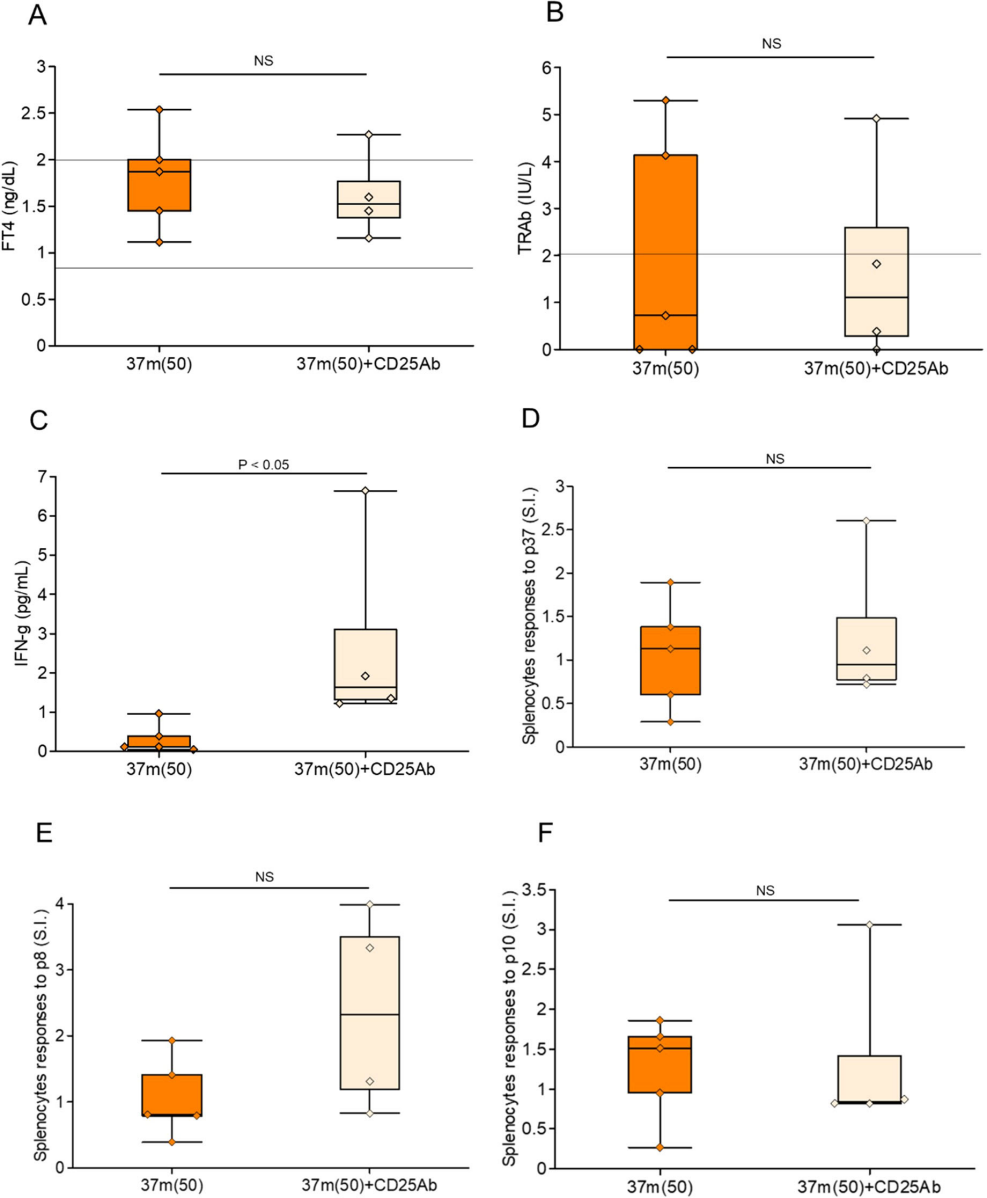
Effects of Treg depletion following step-up immunization with peptide 37m. **(A)** Serum FT4 levels, **(B)** TRAb levels, and **(C)** serum IFN-γ levels were measured at the time of sacrifice, five weeks after Ad-TSH-R289 immunization. **(D–F)** Stimulation index (SI) of splenocytes in response to peptide p37 **(D)**, p10 **(E)**, and p8 **(F)**, as determined by BrdU incorporation assay. Mice were pretreated with step-up immunization using peptide 37m (up to 50 μg), either without (left: 37m [50]) or with (right: 37m [50] + CD25 Ab) administration of anti-CD25 antibody. Treg depletion was achieved by intraperitoneal injection of anti-CD25 antibody four days prior to Ad-TSH-R289 immunization. NS, not significant.

We investigated the role of Tregs in mediating the therapeutic effect of peptide pretreatment in GD (**Figures 5A, B**). Splenic Treg depletion was confirmed by flow cytometric analysis four days after administration of anti-CD25 antibody (**Supplementary Figures 4A–D**). Mice pretreated with the previously identified immunomodulatory peptide 37m were compared to those additionally administered anti-CD25 antibodies prior to Ad-TSH-R289 immunization to deplete Tregs. Administration of anti-CD25 antibodies effectively reduced the Treg population in splenocytes and led to a significant increase in serum IFN-γ levels (P<0.05); however, FT4 and TRAb levels remained unchanged (**Figures 6A–C**). Splenocyte proliferative responses to TSH-R peptides p37, p8, and p10 were not significantly different between the anti-CD25 antibody–treated group and the untreated group (**Figures 6D–F**). Although not statistically significant, the control peptide p8 exhibited a higher stimulation index in the anti-CD25 antibody–treated group (**Figure 6E**).

### Comparison of mice with TRAb positivity

**Figure 7:**
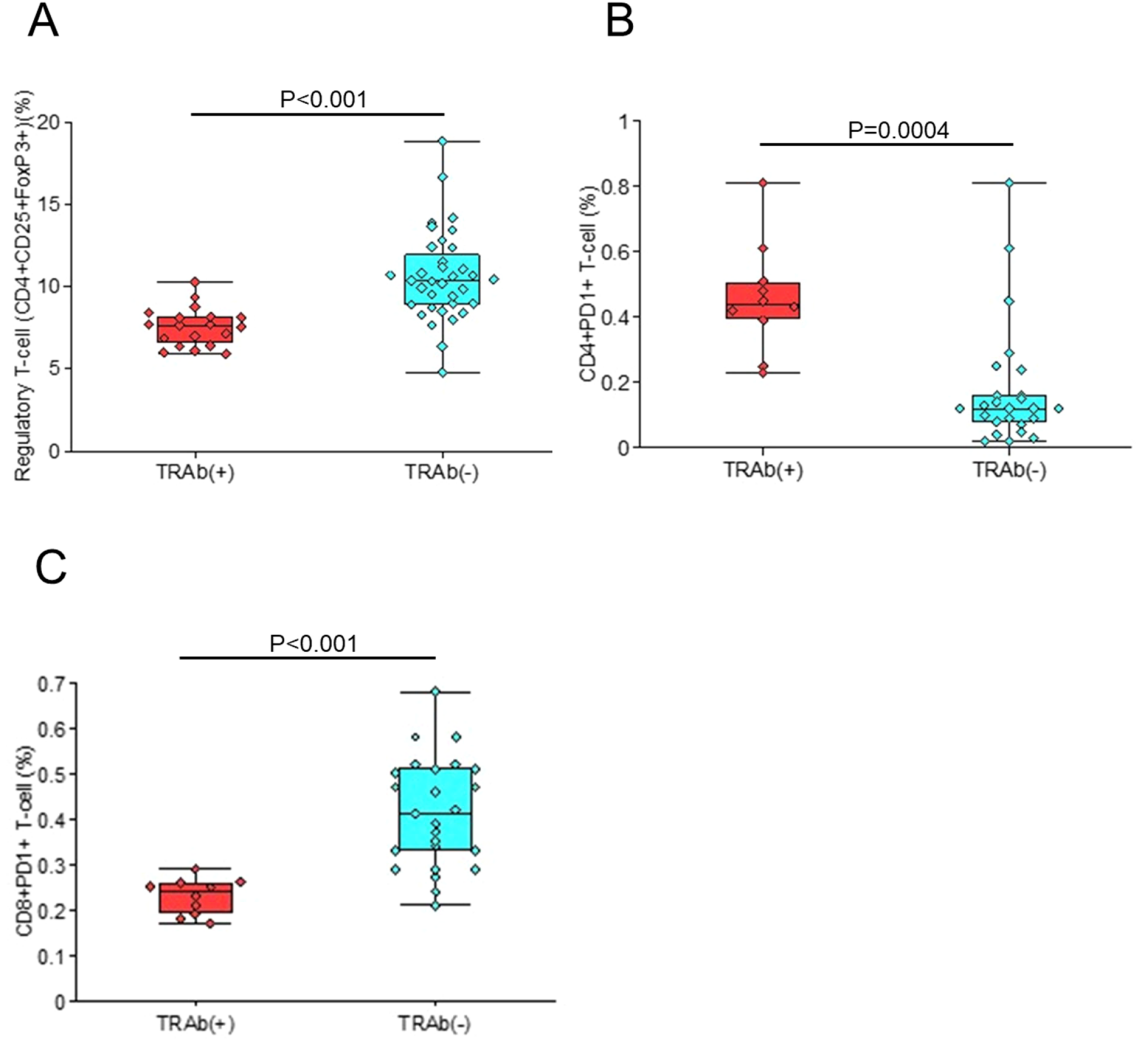
Comparison between TRAb-positive and TRAb-negative mice. The frequencies of splenic Tregs **(A)**, CD4^+^PD-1^+^ T cells **(B)**, and CD8^+^PD-1^+^ T cells **(C)** were examined in mice stratified according to TRAb status.

Finally, mice were divided into TRAb-positive and TRAb-negative groups and analyzed for the frequencies of splenic Tregs, CD4^+^PD-1^+^, and CD8^+^PD-1^+^ T cells (**Figures 7A–C**). TRAb-positive mice exhibited a significantly reduced Treg population (P<0.001) (**Figure 7A**), an increased proportion of CD4^+^PD-1^+^ T cells (P = 0.004) (**Figure 7B**), and a decreased frequency of CD8^+^PD-1^+^ T cells (P<0.001) (**Figure 7C**).

## Discussion

This study demonstrated that pre-immunization with TSH-R–derived peptides can induce antigen-specific immune tolerance and effectively prevent the development of GD in HLA-DR3 transgenic mice. Following immunization with Ad-TSH-R289, a subset of mice developed FT4 elevation, tested positive for TRAb, and exhibited histopathological features consistent with GD. Among the peptides and immunization protocols tested, the step-up administration of p37—escalating up to 50 μg—was the most effective in suppressing increases in serum FT4 and TRAb levels and in preventing GD-associated histological changes. This protective effect was associated with a significant expansion of Tregs, a reduction in CD4^+^PD-1^+^ T cells, and an increase in CD8^+^PD-1^+^ T cells, suggesting a shift toward immune regulation.

Jansson et al. previously established a GD mouse model by immunizing HLA-DR3 or HLA-DR4 transgenic mice with TSH-R, and demonstrated that two TSH-R peptides—similar to the epitopes identified in our earlier studies (7–9)—effectively prevented disease onset (21). Our findings are consistent with these results and further extend them by showing that the native p37 peptide was more effective than its mutated counterpart (37m) in preventing GD. Notably, subcutaneous administration of the peptide dissolved in PBS was sufficient, eliminating the need for complete Freund’s adjuvant (CFA). We also evaluated multiple TSH-R peptides, assessed splenic immune cell subsets including Tregs via flow cytometry, and quantified TRAb titers and serum cytokines. Additionally, Misharin et al. reported that pre-immunization with TSH-R protein can convert stimulatory antibodies into non-functional forms (22), further supporting the concept of antigen-specific modulation of autoimmune responses.

Wu et al. demonstrated that administration of a TSH-R–encoding adenovirus suppressed the onset of neonatal GD and was associated with increased splenic Tregs (23). Consistent with this, our data suggest that the prophylactic effect of p37 is at least partly mediated by Treg expansion. To directly assess the contribution of Tregs, we performed depletion experiments using anti-CD25 antibodies. Treg depletion resulted in a significant reduction in splenic Tregs and a concomitant increase in serum IFN-γ levels. Moreover, some anti-CD25 antibody–treated mice exhibited enhanced proliferative responses to TSH-R peptides, including the control peptide p8, suggesting a partial restoration of autoreactive T-cell responses and epitope spreading (**Figures 6D–F**).

In the single-dose immunization setting, suppression of GD was correlated with the Th1/Th2 cytokine ratio, which in turn was associated with Treg frequency (**Table 2A**). GD is traditionally considered a Th2-skewed disorder (1–3), and our findings suggest that Treg expansion may facilitate a shift toward a Th1-dominant response, thereby contributing to disease suppression. Interestingly, the proportion of naïve T cells was inversely correlated with Treg levels, and appeared to be detrimental. Although the underlying mechanism remains unclear, previous studies suggest that FoxP3 induction in naïve CD4^+^ T cells is inversely related to proliferation, and that excessive naïve T cells may impair Treg differentiation through intraclonal competition (24).

The step-up immunization protocol more effectively induced Tregs and suppressed rebound immune activation compared to the single-dose protocol (**Figures 4A, B**). Mice receiving step-up p37 dosing up to 50 μg exhibited significantly reduced FT4 and TRAb levels, along with decreased CD4^+^PD-1^+^ and increased CD8^+^PD-1^+^ T cells—immune shifts associated with disease suppression. These findings are in line with prior reports showing that elevated CD4^+^PD-1^+^ T cells are associated with autoimmune diseases such as systemic lupus erythematosus (25), while PD-1^+^CD8^+^ T cells play a protective role in multiple sclerosis (26). The reduction in CD4^+^PD-1^+^ and increase in CD8^+^PD-1^+^ T cells may provide insights into new mechanistic hypotheses. We speculate that p37-induced tolerance differentially modulates PD-1 signaling, restraining activated/exhausted CD4^+^PD-1^+^ cells while promoting PD-1^+^CD8^+^ populations with regulatory potential. Further functional validation—such as cytokine profiling or transcriptomic analyses—will be valuable to examine these mechanisms. Together, these findings underscore the immunomodulatory potential of p37. Moreover, the relatively weaker protective effect of the mutated 37m peptide highlights the importance of intact epitope structure in promoting antigen-specific immune tolerance.

Finally, mice positive for TRAb were found to have a reduced Treg population (**Figure 7A**). These findings suggest that a decrease in Tregs is associated with TRAb positivity, and that enhancing the Treg population may help prevent or ameliorate the development of GD. Concerns about persistence of tolerance and potential for epitope spreading remain unresolved. While Treg expansion correlated with protection, partial proliferative responses to non-index peptides were observed, suggesting that minimal diversification cannot be excluded. These issues warrant clarification in future confirmatory studies. From a translational standpoint, several challenges warrant emphasis: (i) dosing feasibility, (ii) HLA diversity, and (iii) safety. Our step-up regimen demonstrates proof-of-concept, but clinical translation will require optimization of peptide dose and interval. Because p37 is restricted by HLA-DRB1*03:01, broader applicability will depend on individualized peptide selection to address HLA diversity. Regarding safety, careful attention should be given to systemic, cutaneous, and immunological aspects, including potential off-target immune modulation. These parameters should be evaluated through comprehensive safety monitoring in future studies.

This study has several limitations. Only female mice were used, as sex-based differences in thyroid hormone levels and immune responses made consistent evaluation challenging. Additionally, adenoviral immunization was performed only once, which may have induced a relatively mild immune response. Nevertheless, these conditions were suitable for detecting subtle immunological changes relevant to tolerance induction. These factors should be considered when interpreting the results and designing future studies. Our observation window was limited to five weeks after disease induction. Although p37 step-up dosing consistently suppressed FT4/TRAb and pathology in the short term, the long-term durability of tolerance remains to be determined. As the current step-up dosage protocol seems to be effective before the development of GD, its applicability after GD onset should be tested, as this would be more clinically relevant.

In conclusion, step-up peptide immunotherapy with the immunodominant TSH-R peptide p37 induced antigen-specific tolerance and effectively prevented GD in HLA-DR3 transgenic mice. These findings demonstrate the feasibility of a TSH-R-based antigen-specific approach for GD, while highlighting the need for further evaluation of durability, HLA coverage, and safety under clinically relevant settings.

## Data Availability

The original contributions presented in the study are included in the article/**Supplementary Material**. Further inquiries can be directed to the corresponding author.

## Supplementary material

Supplementary Figure 1 Representative thyroid histology. **(A)** Thyroid section from a mouse immunized with Ad-TSH-R289, showing colloid enlargement and vacuolization, consistent with thyrotoxicosis and TRAb positivity. **(B)** Thyroid section from a mouse pretreated with a single dose (50 μg) of peptide p37 prior to Ad-TSH-R289 immunization, demonstrating normal histological architecture, with euthyroidism and TRAb negativity.

Supplementary Figure 2 Serum FT4 and TRAb levels in mice treated with Ad-TSH-R289 or Ad-CMV-DEST. (A) Serum FT4 levels, and **(B)** TRAb levels were measured at the time of sacrifice, five weeks after immunization of Ad-TSH-R289 or control adenovirus vector, Ad-CMV-DEST. Reference ranges: FT4, 0.79–2.00 ng/dL; TRAb, <2.0 IU/L (indicated by horizontal lines).

Supplementary Figure 3 Representative flow cytometric analysis of splenic Tregs. **(A)** Flow cytometric analysis of splenic regulatory T cells (Tregs) in a mouse that developed thyrotoxicosis after Ad-TSH-R289 immunization. Lymphocytes were first gated (G1), followed by CD4^+^ T cell gating (G2), and identification of CD25^+^FoxP3^+^ cells within the CD4^+^ population. The percentage of CD4^+^CD25^+^FoxP3^+^ Tregs was 5.8%, representing baseline levels. **(B)** Flow cytometric analysis of splenic Tregs from a mouse pretreated with a single dose (50 μg) of peptide p37 before Ad-TSH-R289 immunization. The proportion of CD4^+^CD25^+^FoxP3^+^ Tregs increased to 9.4%.

Supplementary Figure 4 Depletion of Tregs following anti-CD25 antibody treatment. Flow cytometric analysis of splenic Tregs was performed four days after intraperitoneal administration of either PBS **(A, C)** or anti-CD25 antibody **(B, D)**. The frequency of CD4^+^CD25^+^ T cells decreased from 6.0% **(A)** to 0.4% **(C)**, and that of CD25^+^FoxP3^+^ T cells decreased from 2.8% **(B)** to 0.1% **(D)**. These results confirm a marked depletion of Tregs induced by anti-CD25 antibody treatment.
