# Supplementary figures and images for: Immune tolerance induction using the thyrotropin receptor epitope 78–94 (p37) prevents Graves’ disease in HLA-DR3 transgenic mice
Source: Front Immunol. 2025 Nov 3;16:1633350. doi: 10.3389/fimmu.2025.1633350 (PMC12620236; doi:10.3389/fimmu.2025.1633350)

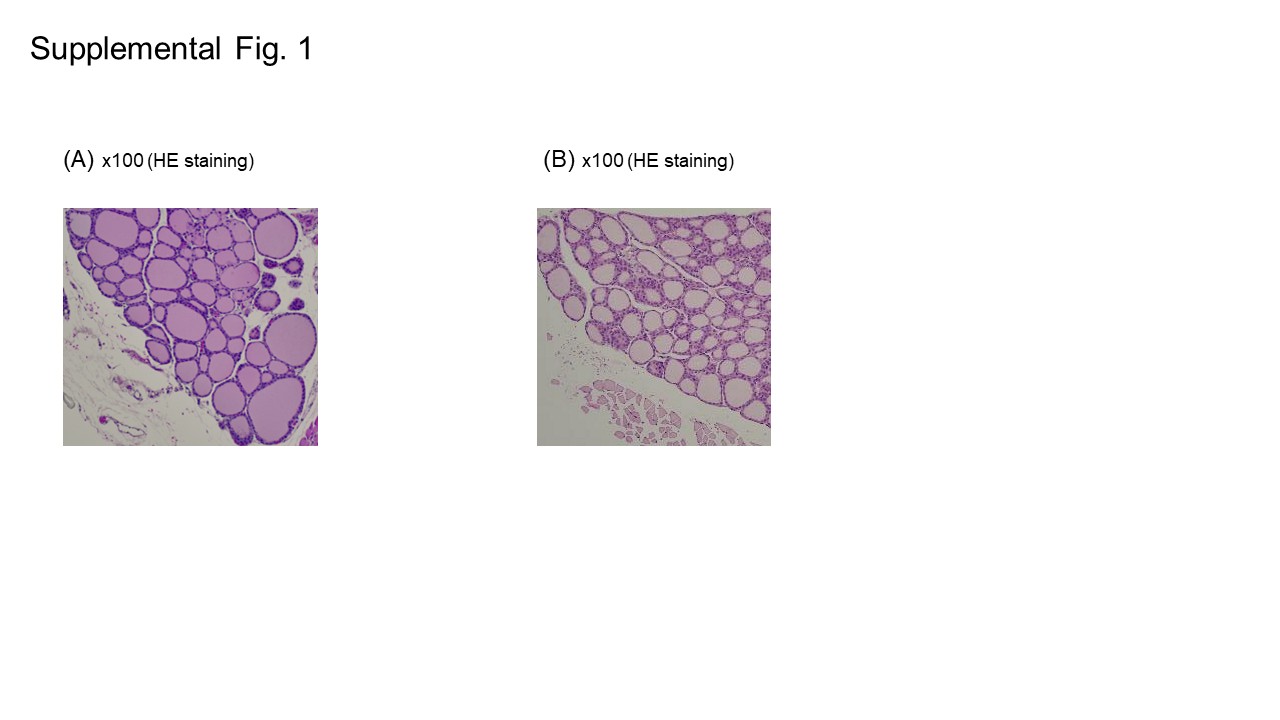

Supplement: Supplementary Figure 1 — Representative thyroid histology. (A) Thyroid section from a mouse immunized with Ad-TSH-R289, showing colloid enlargement and vacuolization, consistent with thyrotoxicosis and TRAb positivity. (B) Thyroid section from a mouse pretreated with a single dose (50 μg) of peptide p37 prior to Ad-TSH-R289 immunization, demonstrating normal histological architecture, with euthyroidism and TRAb negativity. [file Image1.jpeg]

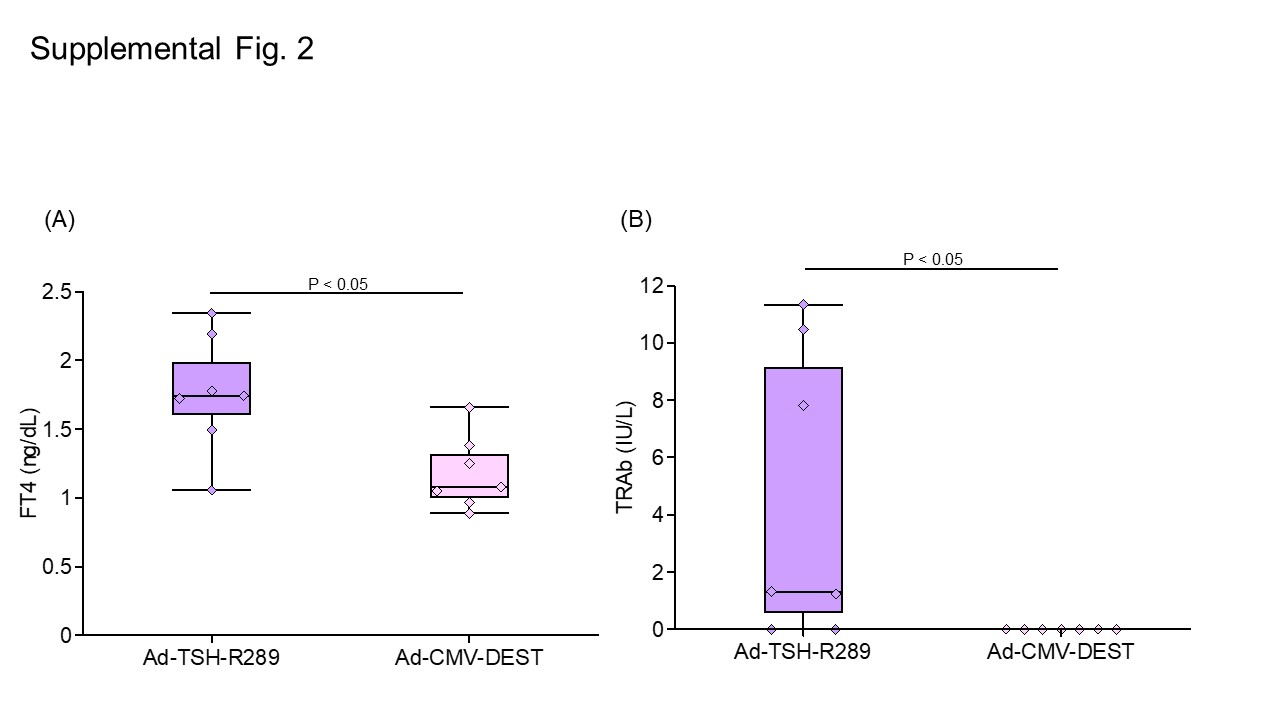

Supplement: Supplementary Figure 2 — Serum FT4 and TRAb levels in mice treated with Ad-TSH-R289 or Ad-CMV-DEST. (A) Serum FT4 levels, and (B) TRAb levels were measured at the time of sacrifice, five weeks after immunization of Ad-TSH-R289 or control adenovirus vector, Ad-CMV-DEST. Reference ranges: FT4, 0.79–2.00 ng/dL; TRAb, <2.0 IU/L (indicated by horizontal lines). [file Image2.jpeg]

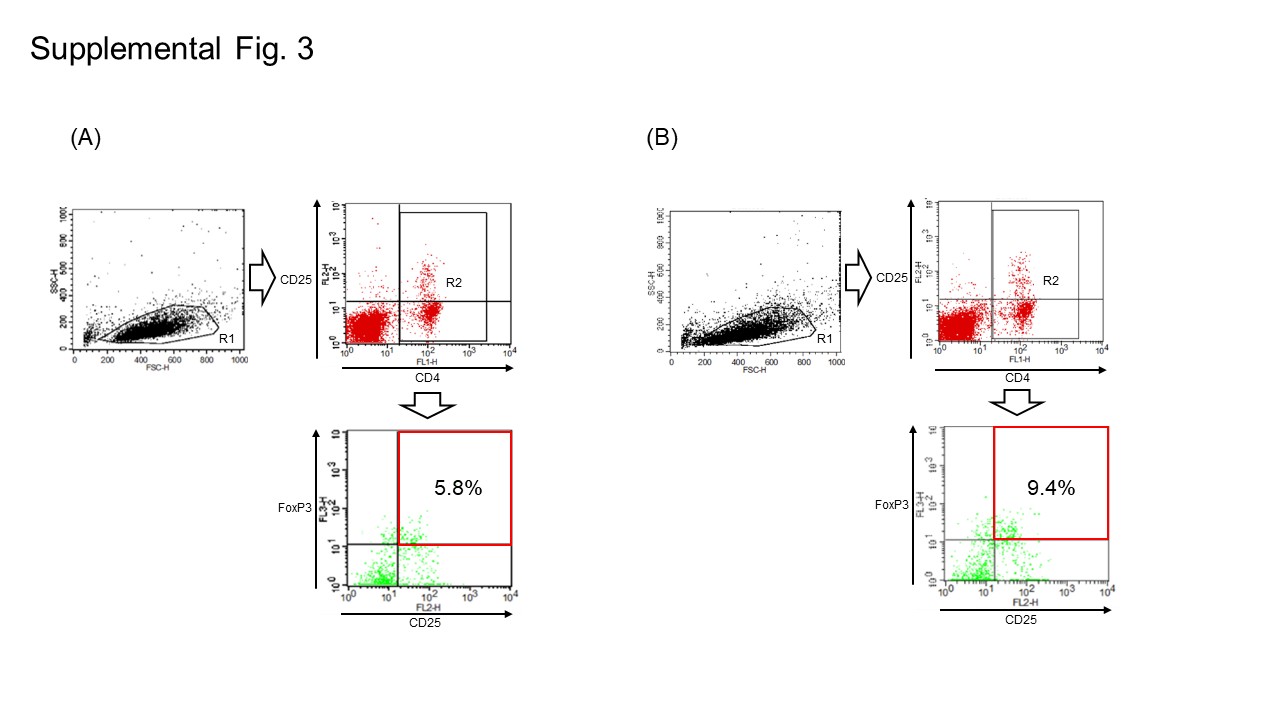

Supplement: Supplementary Figure 3 — Representative flow cytometric analysis of splenic Tregs. (A) Flow cytometric analysis of splenic regulatory T cells (Tregs) in a mouse that developed thyrotoxicosis after Ad-TSH-R289 immunization. Lymphocytes were first gated (G1), followed by CD4+ T cell gating (G2), and identification of CD25+FoxP3+ cells within the CD4+ population. The percentage of CD4+CD25+FoxP3+ Tregs was 5.8%, representing baseline levels. (B) Flow cytometric analysis of splenic Tregs from a mouse pretreated with a single dose (50 μg) of peptide p37 before Ad-TSH-R289 immunization. The proportion of CD4+CD25+FoxP3+ Tregs increased to 9.4%. [file Image3.jpeg]

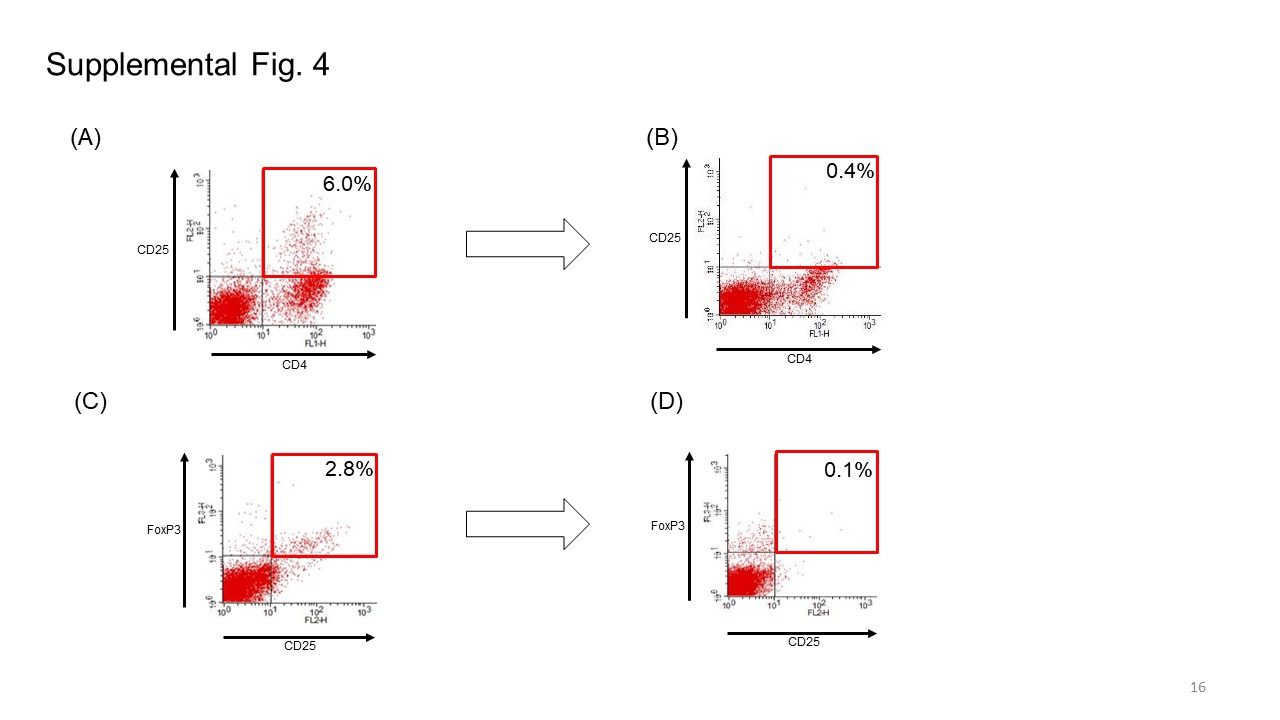

Supplement: Supplementary Figure 4 — Depletion of Tregs following anti-CD25 antibody treatment. Flow cytometric analysis of splenic Tregs was performed four days after intraperitoneal administration of either PBS (A, C) or anti-CD25 antibody (B, D). The frequency of CD4+CD25+ T cells decreased from 6.0% (A) to 0.4% (C), and that of CD25+FoxP3+ T cells decreased from 2.8% (B) to 0.1% (D). These results confirm a marked depletion of Tregs induced by anti-CD25 antibody treatment. [file Image4.jpeg]

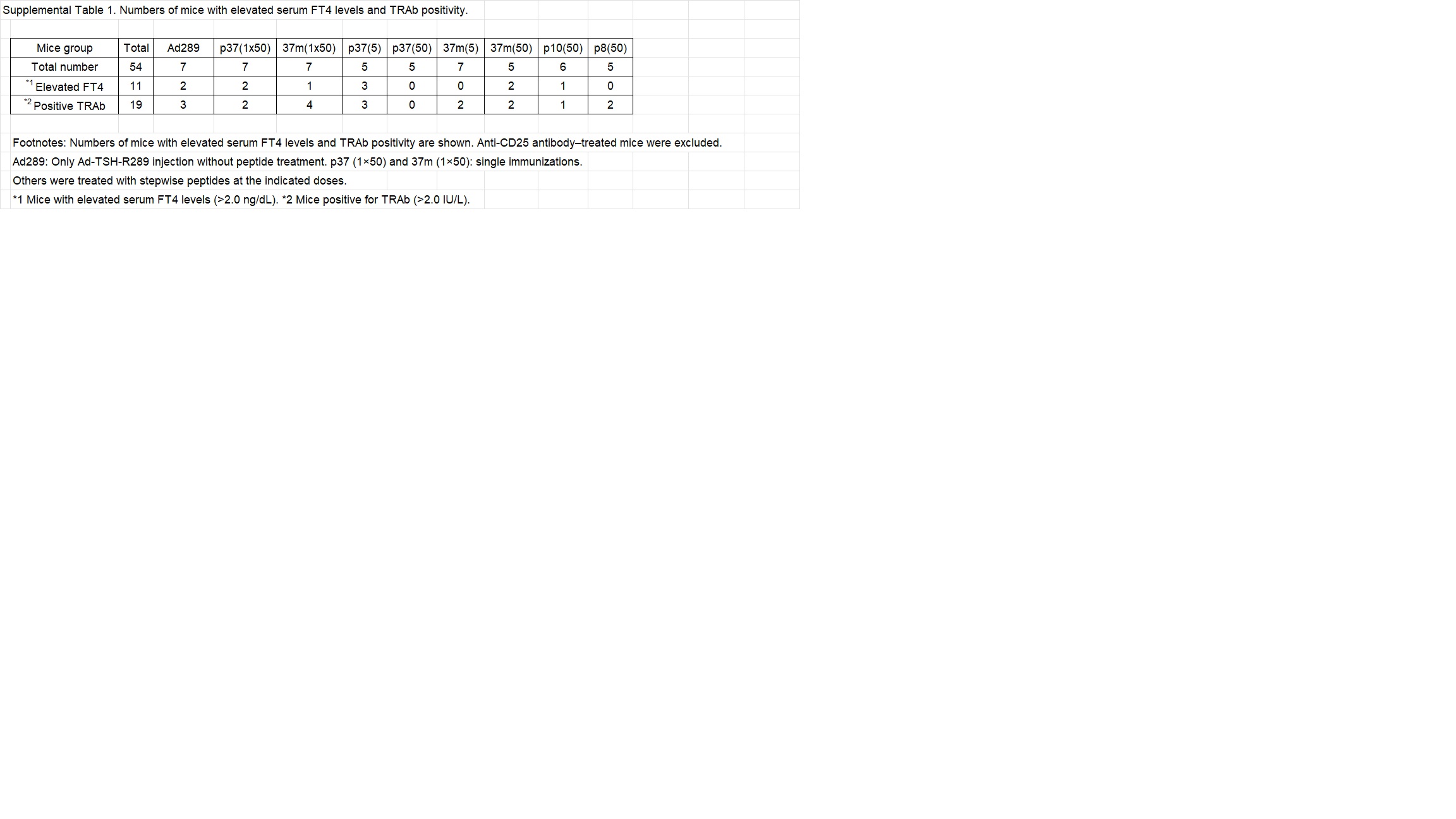

Supplement: Supplementary file 5 [file Image5.jpeg]
